# Update: Increase in Human Infections with Novel Asian Lineage Avian Influenza A(H7N9) Viruses During the Fifth Epidemic — China, October 1, 2016–August 7, 2017

**DOI:** 10.15585/mmwr.mm6635a2

**Published:** 2017-09-08

**Authors:** James C. Kile, Ruiqi Ren, Liqi Liu, Carolyn M. Greene, Katherine Roguski, A. Danielle Iuliano, Yunho Jang, Joyce Jones, Sharmi Thor, Ying Song, Suizan Zhou, Susan C. Trock, Vivien Dugan, David E. Wentworth, Min Z. Levine, Timothy M. Uyeki, Jacqueline M. Katz, Daniel B Jernigan, Sonja J. Olsen, Alicia M. Fry, Eduardo Azziz-Baumgartner, C. Todd Davis

**Affiliations:** ^1^Influenza Division, National Center for Immunization and Respiratory Diseases, CDC; ^2^These authors contributed equally to this report; ^3^China CDC, Beijing; ^4^National Institute for Viral Disease Control and Prevention, China CDC, Beijing; ^5^U.S. CDC, Beijing, China.

Among all influenza viruses assessed using CDC’s Influenza Risk Assessment Tool (IRAT), the Asian lineage avian influenza A(H7N9) virus (Asian H7N9), first reported in China in March 2013,* is ranked as the influenza virus with the highest potential pandemic risk (*1*). During October 1, 2016–August 7, 2017, the National Health and Family Planning Commission of China; CDC, Taiwan; the Hong Kong Centre for Health Protection; and the Macao CDC reported 759 human infections with Asian H7N9 viruses, including 281 deaths, to the World Health Organization (WHO), making this the largest of the five epidemics of Asian H7N9 infections that have occurred since 2013 (Figure 1). This report summarizes new viral and epidemiologic features identified during the fifth epidemic of Asian H7N9 in China and summarizes ongoing measures to enhance pandemic preparedness. Infections in humans and poultry were reported from most areas of China, including provinces bordering other countries, indicating extensive, ongoing geographic spread. The risk to the general public is very low and most human infections were, and continue to be, associated with poultry exposure, especially at live bird markets in mainland China. Throughout the first four epidemics of Asian H7N9 infections, only low pathogenic avian influenza (LPAI) viruses were detected among human, poultry, and environmental specimens and samples. During the fifth epidemic, mutations were detected among some Asian H7N9 viruses, identifying the emergence of high pathogenic avian influenza (HPAI) viruses as well as viruses with reduced susceptibility to influenza antiviral medications recommended for treatment. Furthermore, the fifth-epidemic viruses diverged genetically into two separate lineages (Pearl River Delta lineage and Yangtze River Delta lineage), with Yangtze River Delta lineage viruses emerging as antigenically different compared with those from earlier epidemics. Because of its pandemic potential, candidate vaccine viruses (CVV) were produced in 2013 that have been used to make vaccines against Asian H7N9 viruses circulating at that time. CDC is working with partners to enhance surveillance for Asian H7N9 viruses in humans and poultry, to improve laboratory capability to detect and characterize H7N9 viruses, and to develop, test and distribute new CVV that could be used for vaccine production if a vaccine is needed.

**FIGURE 1:**
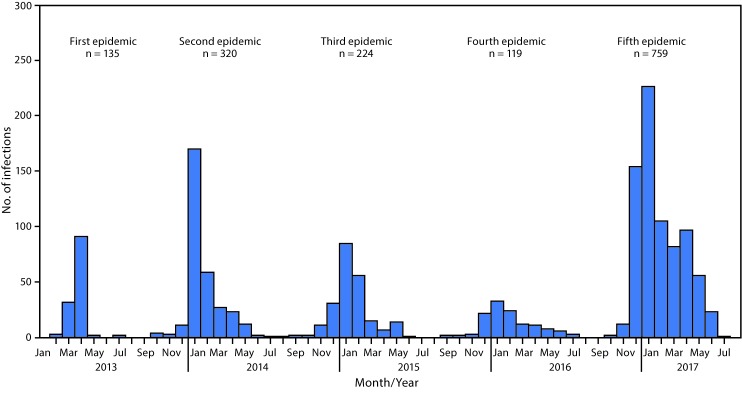
Confirmed Asian lineage avian influenza A(H7N9) virus infections of humans reported to the World Health Organization (N = 1,557),* by month of illness onset — China,† February 19, 2013–August 7, 2017 **Source:** Publically released infections in Disease Outbreak News (http://www.who.int/csr/don/en/) or Human-Animal Interface Monthly Report (http://www.who.int/influenza/human_animal_interface/en/). * Date of onset missing for six infections. ^^†^^ One case was exported to Malaysia (January 2014) and two to Canada (January 2015).

Epidemiologic data were collected from the WHO Disease Outbreak News^†^ and Influenza Risk Assessment summaries,^§^ and from recent publications. Genetic and virus characterization data were collected from the publically accessible Global Initiative on Sharing All Influenza Data and GenBank databases.^¶^ Nucleotide sequence alignments of hemagglutinin (HA) and neuraminidase (NA) genes were created and used to generate phylogenetic trees for lineage determination. Amino acid changes in fifth-epidemic viruses were identified through comparisons with CVVs produced using 2013 Asian H7N9 virus sequences, and identification of viruses as either LPAI or HPAI was accomplished by analysis of the HA cleavage site. CDC assessed the antigenic properties of virus isolates using the hemagglutination inhibition (HI) assay employing a panel of reference ferret antisera that includes antisera raised to LPAI Yangtze River Delta and Pearl River Delta fifth-epidemic viruses, HPAI H7N9 viruses, and the 2013 CVVs. The extent of cross-reactivity with 2013 CVVs and viruses from previous epidemics was assessed.

## Epidemiology

**FIGURE 2:**
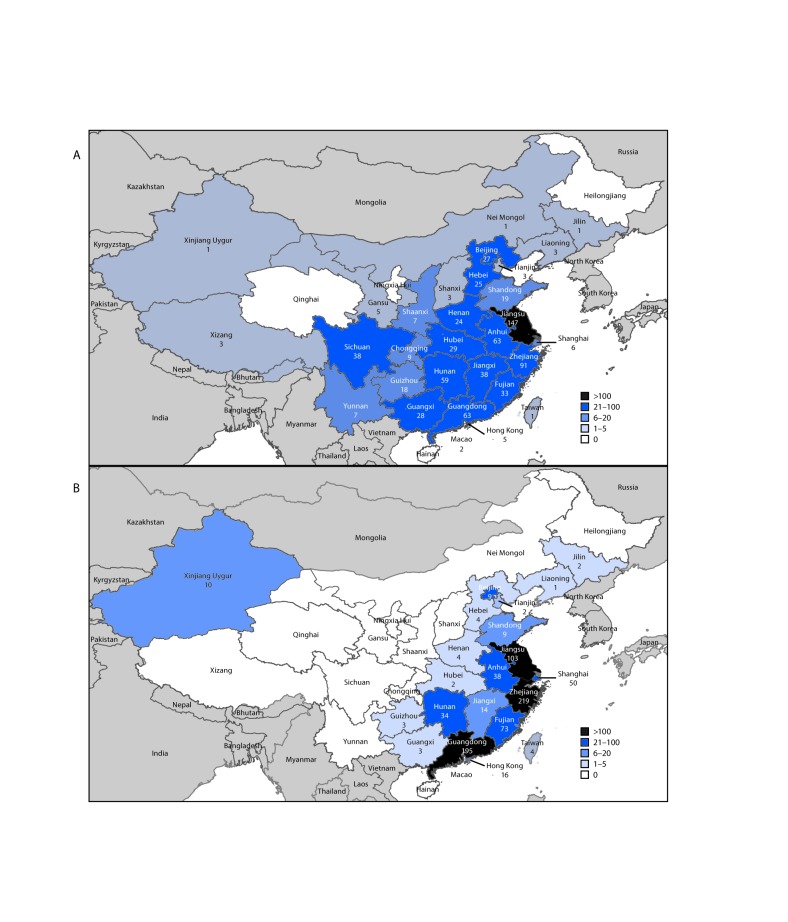
Geographic distribution of Asian lineage avian influenza A(H7N9) virus infections of humans reported to the World Health Organization — China,* A) epidemic 5 (October 1, 2016–August 7, 2017) and B) epidemics 1–4 (March 2013–September 30, 2016) **Source:** Publically released infections in Disease Outbreak News (http://www.who.int/csr/don/en/) or Human-Animal Interface Monthly Report (http://www.who.int/influenza/human_animal_interface/en/). * Avian influenza A(H7N9) virus infections of humans reported in mainland China, Hong Kong, Macao, and Taiwan.

During March 31, 2013–August 7, 2017, a total of 1,557 human infections with Asian H7N9 viruses were reported; at least 605 (39%) of these infections resulted in death. All infections were either detected in mainland China, Hong Kong, and Macao, or associated with travel from mainland China (29 cases were exported to Malaysia, Canada, Hong Kong, Macao, and Taiwan). Infections were reported from more provinces, regions, and municipalities in China during the fifth epidemic (30) than during the first four epidemics combined (21) (Figure 2). Similar to epidemics 1–4, 70% of infections during the fifth epidemic occurred in men, and the median age was 57 years (range = 4–93 years); most occurred among persons with recent poultry exposure (90%) and resulted in severe respiratory illness (90%)** (*2*). Among the 759 reported infections during the fifth epidemic, 14 clusters of two or three persons with Asian H7N9 virus infections were reported to WHO, compared with an average of nine clusters in each of the previous epidemics (range = 4–11 clusters).

During the fifth epidemic, LPAI Asian H7N9 viruses acquired an HPAI mutation that causes increased morbidity and mortality in poultry. Ten of 33 provinces, regions, and municipalities in China reported HPAI Asian H7N9 viruses in poultry and environmental samples: Fujian, Guangdong, Guangxi, Hebei, Heilongjiang, Henan, Hunan, Inner Mongolia, Shaanxi, and Tianjin.^††^ Among the 759 human infections identified in the fifth epidemic, 27 were HPAI Asian H7N9 viruses (11 from Guangxi, eight from Guangdong, five from Hunan, one from Hebei and one from Shaanxi Provinces, and one from Taiwan)^§§^ (*3*). Human infections with HPAI Asian H7N9 viruses were significantly more likely to occur in rural areas, among persons with early hospital admission, and after exposure to sick or dead poultry, but were otherwise similar in their demographic and clinical characteristics to infections with LPAI Asian H7N9 viruses (*3*,*4*).

## Analysis of Virus Gene Sequences

Analysis of HA gene sequences demonstrated two distinct Asian H7N9 virus lineages isolated from humans during the fifth epidemic: the Pearl River Delta lineage and Yangtze River Delta lineage (*5*). Among 166 fifth-epidemic Asian H7N9 virus HA gene sequences entered into Global Initiative on Sharing All Influenza Data and GenBank, 160 were from infected humans in mainland China, five were from Hong Kong, and one was from Taiwan. A total of 159 of the virus HA sequences were from the Yangtze River Delta lineage and seven were from the Pearl River Delta lineage, indicating the predominance of the Yangtze River Delta lineage. In addition, 35 (21%) of the 166 fifth-epidemic Asian H7N9 virus specimens and samples (27, 77% from human specimens and 8, 23% from environmental samples) with publicly available sequences had a four amino acid insertion in the cleavage site of the HA protein indicating a mutation found in HPAI viruses (*6*).

NA gene sequence data were available for all 166 viruses collected during the fifth epidemic; 18 (10.8%) viruses (all from patients who were possibly treated with NA inhibitors) had genetic markers of reduced susceptibility to one or more NA inhibitors. All 166 viruses had the S31N mutation in the M2 protein, indicating resistance to amantadine and rimantadine, as was observed in previous Asian H7N9 epidemics (*7*).

## Analysis of Virus Surface Proteins

HI testing of fifth-epidemic Yangtze River Delta lineage viruses, which accounted for the majority of available viruses, demonstrated significant antigenic differences compared with 2013 CVVs produced from 2013 Asian H7N9 viruses.^¶¶^ Ferret antisera raised against the 2013 CVVs poorly inhibited hemagglutination of fifth-epidemic Yangtze River Delta lineage viruses compared with inhibition of viruses tested from previous epidemics. HI testing of HPAI Asian H7N9 viruses also indicated significant antigenic differences compared with 2013 CVVs. As part of a National Institutes of Health trial, sera from adults who received vaccine produced using a 2013 Asian H7N9 CVV showed reduced cross-reactive HI and neutralizing antibody titers to fifth-epidemic Yangtze River Delta lineage and HPAI viruses, compared with titers to 2013 H7N9 viruses (*8*).

## Discussion

The fifth annual epidemic of Asian H7N9 in China is marked by extensive geographic spread in poultry and in humans. The number of human infections reported in the fifth epidemic is almost as many as were reported during the previous four epidemics combined. The consistent epidemiology combined with a similar number of clusters suggests that the increased number of human infections appears to be associated with wider geographic spread and higher prevalence of Asian H7N9 viruses among poultry rather than any increased incidence of poultry-to-human or human-to-human spread. Furthermore, surveillance and testing have remained relatively unchanged from the fourth to fifth epidemic.

Although human infections with Asian H7N9 viruses from poultry are rare and no efficient or sustained human-to-human transmission has been detected, when human infections do occur, they are associated with severe illness and high mortality. Continued vigilance is important to identify changes in the virus that might have epidemiologic implications, such as increased transmission from poultry to humans or transmission between humans.

CDC assesses the pandemic potential of novel influenza A viruses using the IRAT evaluation tool. The IRAT analysis process considers the properties of the specific virus, attributes of the population, and associated ecology and epidemiology to assess the potential pandemic risk posed to human health by each virus. In light of the increased number of human infections and virologic changes observed during the fifth epidemic, CDC carried out an IRAT assessment of the newly emerged Yangtze River Delta lineage LPAI Asian H7N9 virus. This virus lineage scored as having the highest potential pandemic risk (moderate to high) among viruses similarly evaluated using the IRAT (*1*).

In March 2017, WHO recommended the development of new CVVs to match the antigenically distinguishable Yangtze River Delta viruses. The WHO Collaborating Center for the Surveillance, Epidemiology and Control of Influenza at CDC generated a new Asian H7N9 CVV derived from a Yangtze River Delta lineage LPAI Asian H7N9 virus, A/Hong Kong/125/2017 (an A/Hunan/02650/2016-like virus) (*9*). The WHO Collaborating Center for Reference and Research on Influenza in China developed a CVV from an HPAI Asian H7N9 virus, A/Guangdong/17SF003/2016. These CVVs, as well as others being developed by other WHO Collaborating Centers for Influenza, can be used for vaccine production, clinical trials, stockpiling and other pandemic preparedness purposes based on ongoing public health risk assessment.

The Government of China remains committed to controlling the transmission of Asian H7N9 viruses from birds to humans, and to mitigating human infections. Specific control measures implemented by the Government of China in response to the pandemic threat include strategies to minimize spread through promoting large-scale farming and centralized slaughtering, improving poultry product cold chain transportation and storage at markets, routine live poultry market closures with cleaning and disinfection, and a national poultry vaccination program (*10*). To monitor and control human infections, the Government of China has issued prevention and control protocols that include guidance on enhanced surveillance for influenza-like illness, severe acute respiratory infection, and pneumonia of unknown etiology; case investigation and contact tracing; and diagnosis and treatment guidance. Additional strategies to monitor and control human infections include conducting field investigations to identify and monitor close contacts of confirmed human infections, and testing environmental samples from possible exposure locations such as live bird markets (*10*).

The findings in this report are subject to at least three limitations. First, underreporting of human infections and deaths with Asian H7N9 viruses is likely, given that most are identified through a passive surveillance system. Second, delays between what has been officially reported to, and publically released by, WHO might occur; thus numbers in this report might vary from those reported by other sources. Finally, as more genetic and antigenic data become available, further evaluation and characterization of these viruses might reveal additional differences.

The evolving Asian H7N9 viruses highlight the importance of rapid analysis and public sharing of sequence data to inform pandemic preparedness efforts. These data allow for the rapid identification of genetic changes known to be associated with antigenic variation, antiviral drug susceptibility, mammalian adaptation, virulence and transmissibility. Assessments based on sequence data have the potential to inform surveillance, guide allocation of outbreak response resources, and inform pandemic preparedness policy decisions, such as selecting viruses for CVV development, and purchasing of prepandemic vaccines and antivirals. CDC continues to partner with China CDC, together with other China government organizations, United Nations organizations, and the governments of surrounding countries to support surveillance for Asian H7N9 viruses in humans, poultry, and environmental samples from live bird markets, and to enhance laboratory capacity. CDC’s International Influenza Program also supports efforts by >50 countries to detect and respond to novel influenza A virus threats.*** Guidance for travelers to China is provided at the U.S. CDC Travelers’ Health website, (https://wwwnc.cdc.gov/travel/notices/watch/avian-flu-h7n9).

**Summary**

**What is already known about this topic?**

The current Asian lineage avian influenza A(H7N9) virus (Asian H7N9) epidemic in China is the fifth and largest epidemic on record.

**What is added by this report?**

Human infections with Asian H7N9 virus were reported from more provinces, regions, and municipalities in China during the fifth epidemic than in the previous four epidemics combined. Because of antigenic variation between the Yangtze River Delta lineage viruses, the newly emerged high pathogenic Asian H7N9 viruses, and 2013 candidate vaccine viruses, new candidate vaccine viruses have been produced.

**What are the implications for public health practice?**

These candidate vaccine viruses, as well as others being developed by other World Health Organization Collaborating Centers for Influenza, could be used for vaccine production, clinical trials, stockpiling, and other pandemic preparedness purposes, based on ongoing public health risk assessment. CDC has partnered with China CDC, and other China government organizations, United Nations organizations, and surrounding countries to enhance surveillance and laboratory capacity to detect and respond to Asian H7N9 in animals and humans.

## Footnotes

* Total number of fatal cases in mainland China are published on a monthly basis by China National Health and Family Planning Commission. http://www.nhfpc.gov.cn/jkj/s3578/201706/99b1482bfd7e499db90b3ee133e56e13.shtml.

†
http://www.who.int/csr/don/en/ .

§
http://www.who.int/influenza/human_animal_interface/Influenza_Summary_IRA_HA_interface_06_15_2017.pdf?ua=1 .

¶ Global Initiative on Sharing All Influenza Data (https://www.gisaid.org/). GenBank databases (https://www.ncbi.nlm.nih.gov/genbank/).

**
http://www.who.int/influenza/human_animal_interface/HAI_Risk_Assessment/en/ .

†† http://www.oie.int/wahis_2/public/wahid.php/Diseaseinformation/WI.

§§
http://ivdc.chinacdc.cn/cnic/en/Surveillance/WeeklyReport/201708/t20170828_151326.htm .

¶¶ http://apps.who.int/iris/bitstream/10665/254827/1/WER9212.pdf?ua=1.

*** https://www.cdc.gov/flu/pdf/international/program/2014-15/2014-15-report_background.pdf.
