# *Erratum*: Vol. 66, No. 35

**DOI:** 10.15585/mmwr.mm6640a8

**Published:** 2017-10-13

In the report “Update: Increase in Human Infections with Novel Asian Lineage Avian Influenza A(H7N9) Viruses During the Fifth Epidemic — China, October 1, 2016–August 7, 2017,” on page 929, the last sentence of the first paragraph of the epidemiology section should have read “Among the 759 reported infections during the fifth epidemic, 14 clusters of two or three persons with Asian H7N9 virus infections were reported to WHO, compared with an average of **6.5** clusters in each of the previous epidemics (range = 4–11 clusters).”

